# Aspidosperma species (Apocynaceae) as sources of antimalarials: from the *in vitro* antiplasmodial activity of extracts to pre-clinical toxicological studies for the development of efficient and safe antimalarial phytomedicines

**DOI:** 10.1186/1475-2875-13-S1-P18

**Published:** 2014-09-22

**Authors:** Alaide Braga de Oliveira, Renata Cristina de Paula, Maria Fani Dolabela, Fabiola Rocha, Fabiana Gomes, Giovanny Garavito, Geraldo Célio Brandão, Fúlvio Mendes, Ricardo Tabach

**Affiliations:** 1 Universidade Federal do Pará - UFPA, Belem, Pará, Brazil; 2 Universidade Federal de Minas Gerais - UFMG, Belo Horizonte, Minas Gerais, Brazil; 3 Universidade Federal de Juiz de Fora - UFJF, Juiz de Fora, Minas Gerais, Brazil; 4 Universidad Nacional de Colombia - UNAL, Bogotá, Colombia; 5 Universidade Federal do ABC - UFABC, São Paulo, São Paulo, Brazil; 6 Universidade Federal de Ouro Preto - UFOP, Ouro Preto, Minas Gerais, Brazil; 7 Universidade Federal do Estado de São Paulo - UNIFESP, São Paulo, São Paulo, Brazil

## 

We report here our investigations on plants belonging to the botanical genus Aspidosperma (Apocynaceae) that are used to treat malaria and/or fevers in Brazil, aiming to prepare standardized alkaloid extracts for phytomedicines development. Ethanol and alkaloid extracts of *A. parvifolium* and *A. subincanum* barks were prepared and their *in vitro* antiplasmodial activity was evaluated against *Plasmodium falciparum* (W2 strain) by the pLDH method. Bioguided fractionation of the active alkaloid extracts was undertaken. A study on antiplasmodial activity versus alkaloids content variation from three *A. subincanum* specimens that were collected in different regions was carried on by HPLC-UV. Ethanol and alkaloid extracts from *A. parvifolium* and *A. subincanu*m were evaluated in toxicological studies in male and female Swiss mice and male Wistar rats (n = 10); a control group was included in the experiments. At the end of treatment, animals were sacrificed, hematology and serum biochemical analyses were performed. Fractionation of alkaloid extracts from *A. parvifolium* and *A. subincanum* guided by *in vitro* assays against *P. falciparum* (W2 strain) led to the isolation of the indole alkaloids uleine (Oliveira *et al.*, 2010) and N-demethyluleine. HPLC-UV and LC-UV-MS/ESI analyses of the extracts disclosed uleine as the major constituent, its content reaching 70.7% in the alkaloid extract from *A. subincanum* (ASALB) which was more potent than uleine and N-demethyluleine themselves in the *in vitro* assays, as can be inferred from the selectivity indexes in relation to HepG2 cells (SI = CC_50_/IC_50_) of ASALB (100), uleine (44) and N-demethyluleine (21). Moreover, a significative variation on the contents of these two alkaloids was observed for the same plant species when collected in different regions (Paula, 2014). Both ethanol and alkaloid extracts from *A. parvifolium* bark were relatively safe, producing minor changes in the parameters evaluated, especially in smaller doses, thus making feasible their future use in phase I clinical trials. On the other hand, high toxicity of both extracts from *A. subincanum* bark was observed in doses >200 mg/kg. The present results point to the necessity of rigorous investigation of traditional antimalarial plants including *in vitro* studies for identification of active natural products, standardization of extracts to be submitted to pre-clinical toxicological studies and controlled clinical trials. Our results disclose that efficient and safe phytomedicines might be developed from standardized crude or semi-purified extracts of *Aspidosperma* species containing the active chemical entities.

